# Assessing the Feasibility of Using Apple Vision Pro While Performing Medical Precision Tasks: Controlled User Study

**DOI:** 10.2196/73574

**Published:** 2025-08-15

**Authors:** Hamraz Javaheri, Vitor Fortes Rey, Paul Lukowicz, Gregor A Stavrou, Jakob Karolus, Omid Ghamarnejad

**Affiliations:** 1 Department of Embedded Intelligence, German Research Centre for Artificial Intelligence, Trippstadter Str. 122, Kaiserslautern, 67663, Germany, 49 631 20575 4190; 2 Department of Computer Science, Rheinland-Pfälzische Technische Universität Kaiserslautern-Landau, Kaiserslautern, Germany; 3 Department of General, Visceral, and Oncological Surgery, Klinikum Saarbrücken, Saarbrücken, Germany

**Keywords:** Apple Vision Pro, HoloLens, extended reality, precision task, head-mounted display

## Abstract

**Background:**

The emergence of next-generation video-see-through head-mounted displays, such as the Apple Vision Pro (AVP), has generated considerable interest in the medical field. While preliminary studies highlight AVP’s potential, no controlled study has rigorously assessed its usability for precision-based medical tasks requiring fine motor control and real-world perception.

**Objective:**

This study aims to evaluate the feasibility of using AVP while performing real-world medical precision tasks.

**Methods:**

To assess AVP’s feasibility, we conducted a controlled user study with 20 health care professionals, who performed 3 different suturing techniques across 3 intervention conditions. Participants completed the same tasks using AVP, the Microsoft HoloLens 2 (MHL2), and a baseline (without a head-mounted display). A within-subject design was used, ensuring that each participant experienced all intervention groups. We used a mixed methods research approach, incorporating both quantitative metrics, including task completion time, suturing performance, system usability score, cognitive load, virtual reality sickness, and presence score, as well as qualitative insights gathered through interviews.

**Results:**

Participants took significantly longer to complete the entire task using AVP (570.0, SD 192.0 s) compared with MHL2 (456.0, SD 120.0 s; *P*<.001) and baseline (472.0, SD 143.0 s; *P*<.001). The analysis on participants’ average suture performance revealed no significant differences across interventions (*P*=.76). The total raw NASA Task Load Index score among participants was significantly higher for AVP (43.9, SD 15.9) compared with MHL2 (21.5, SD 13.8; *P*<.001) and baseline (19.1, SD 15.1; *P*<.001). The analysis of the presence questionnaire demonstrated a significantly higher presence score for MHL2 (115.0, SD 11.4) compared with AVP (93.7, SD 12.7; *P*<.001). The overall virtual reality sickness questionnaire score was significantly higher for AVP (66.9, SD 19.8) compared with MHL2 (41.1, SD 9.32; *P*<.001). Moreover, the calculated system usability score for MHL2 (72.7, SD 8.54) was significantly higher compared with AVP (50.3, SD 14.4; *P*<.001).

**Conclusions:**

In conclusion, AVP has potential for non–time-sensitive medical applications or those that emphasize digital elements over real-world interaction. Its current usability limitations, particularly increased cognitive load and prolonged task execution times, suggest that further optimizations are necessary before widespread clinical adoption is feasible.

## Introduction

Mixed reality (MR) has been a transformative technology for several years, revolutionizing various industries and applications. As part of the broader spectrum of immersive technologies [1], MR bridges augmented reality (AR), which overlays digital content onto the real world, and virtual reality (VR), which provides fully immersive digital environments. With recent advancements in wearable technology and head-mounted displays (HMDs), MR has expanded into a wide range of daily activities and professional domains [2]. A significant development in this field is the rise of extended reality (XR) devices, which integrate both AR and VR capabilities, enabling seamless transitions between immersive and real-world experiences. This new generation of devices, such as the Apple Vision Pro (AVP) [3], has sparked considerable interest and is believed to be the future of HMDs in the medical domain, offering users immersive XR experiences through video-see-through (VST) technology [4-12]. Although there are studies that have examined XR applications across various settings and domains, the choice of device and technology for specific task groups is often driven by the latest market trends rather than an informed assessment of their feasibility for the intended use case.

Egger et al [4,5] regarded AVP as a major step toward achieving the “ultimate display” for health care. They highlighted its potential to address challenges that previous MR devices, such as the Microsoft HoloLens (MHL) [13], encountered in terms of precision, reliability, usability, workflow integration, and user perception. Similarly, Masalkhi et al [6] postulated that Apple XR technology holds a wide range of possibilities in ophthalmology, including applications in surgical training, assistive devices, diagnosis, and education. Furthermore, Olexa et al [7] reported using AVP as a neurosurgical planning tool to visualize 3D models of patients. They noted that users found the 3D models to be highly realistic (Likert score of 4.5/5), the real-world view displayed through the headset to be natural (Likert score of 4.3/5), and experienced minimal eye strain or fatigue while using the device.

While these studies highlight the significant potential of AVP in the medical field and its ability to support the development of various beneficial applications, no controlled or experimental study has rigorously evaluated the usability of the device itself and its impact on user real-world performance and experience, independent of the application used. In particular, the suitability of AVP and its VST design remains unexplored in scenarios that involve wearing the headset while performing real-world, delicate tasks requiring fine motor control and precision, such as those encountered in surgical support and navigation systems. As immersive technologies continue to evolve, the extent to which the choice of device directly influences user performance remains unclear. Furthermore, while VST devices, such as AVP, are expected to enhance the accuracy and precision required for medical tasks by providing higher-quality visualization and more robust registration of digital objects, the usability of these technologies and the devices themselves for applications requiring real-world precision remains uncertain, as users rely on visual information through a video stream rather than direct visual perception. Consequently, the safety and feasibility of AVP in critical applications, such as in situ surgical navigational systems, need to be investigated. Additionally, its proclaimed superiority over existing HMDs in medical domains, which demand high levels of accuracy and dexterity, remains unproven. This gap presents an important opportunity for further research to substantiate the benefits and advantages of different XR approaches, including optical see-through (OST) and VST.

Many of the applications envisioned for AVP have already been achieved using MHL, an OST MR device series that has demonstrated a broad range of applications in medicine. These include patient data visualization [14], patient education [15], assistance and monitoring [16], preoperative diagnosis [17], anatomy learning [18,19], intervention training [20], image-guided interventions [21], in situ surgical navigation [22-26], and telemedicine [27]. Extensive use has demonstrated the effectiveness of MHL in these areas and provides a strong baseline for evaluating new XR devices, such as AVP, particularly for medical applications that rely on MR capabilities rather than pure VR and require visual perception of the real world.

This study addresses these gaps by evaluating the feasibility of AVP for performing medical precision tasks that require visual perception of the real world. The main goal of this study is to evaluate the feasibility of the devices and their underlying technology without any bias from specific applications. This work contributes to understanding the impact of device choice on user performance by examining both objective performance metrics and subjective measures of user experience. To achieve this, we designed a controlled user study involving 20 health care professionals, comparing AVP against a baseline (without an HMD) and an extensively used MR glass in the medical domain [14-22,24,25,28], MHL2, for performing 3 different suturing techniques. Our evaluation included both quantitative data, including the system usability score, task completion time (TCT), suturing performance, cognitive load, VR simulation sickness, and presence score, as well as qualitative data gathered through interviews.

## Methods

### Study Design and Protocol

This study adopts mixed methods research methodology, combining both quantitative and qualitative data collection approaches. Three interventions were designed, corresponding to the 3 conditions of the study: baseline (no HMD), MHL2, and AVP. The baseline (no HMD) condition was included to serve as a reference point for evaluating the effects of the other interventions. MHL2 was used due to its extensive prior use in the medical domain. A within-subject design was used, meaning that each participant participated in all studied interventions. The order of participation in each intervention was counterbalanced to mitigate potential order bias.

The study began with an introduction phase involving obtaining informed consent and an introduction to the study tasks from all participants. Following consent, the entire session was recorded using 2 cameras, one egocentric and one exocentric, with front-facing views. Later, participants were randomly assigned to 1 of 6 possible orders for performing the 3 interventions (baseline, MHL2, and AVP). Prior to task execution, participants completed 2 questionnaires on demographics and affinity for technology interaction [29].

Following completion of the preparation and order assignment, the experiment task began. During this stage, participants were asked to perform the same study task, which involved working with 3 different suture types, for each intervention. Prior to performing the study task with AVP and MHL2, calibration procedures were conducted. For MHL2, only eye calibration was performed. For AVP, both eye and hand calibration were performed to address any potential issues arising from lens misalignment or visual discrepancies.

After completing the task for each intervention, photographs of the participants’ performance using the suturing kit were captured for subsequent evaluation. Additionally, web-based questionnaires were administered to evaluate key factors related to the user experience. Cognitive workload was assessed using NASA Task Load Index (NASA-TLX) [30], VR-induced sickness was measured with the virtual reality sickness questionnaire (VRSQ) [31], and the sense of presence in the digital environment was evaluated using the presence questionnaire (PQ) [32]. Furthermore, for a rapid and reliable assessment of new health care technologies [33], UMUX-Lite questionnaire was used. System usability score was then predicted using a regression equation based on the 2 UMUX-Lite items [34]. The order of questions was presented in a random order for each participant. After completing the task for the baseline condition (without an HMD), participants completed only the NASA-TLX [30]. Following the MHL2 and AVP interventions, participants filled out the NASA-TLX, VRSQ, PQ, and UMUX-Lite.

Finally, at the end of each session, a researcher conducted a short semistructured interview with the participants, asking them to reflect on their experience with each device. The interview questions encapsulated aspects including comfort, self-performance evaluation, pros and cons, and potential use cases.

After all data recording sessions were completed, the TCTs were extracted from the recorded videos. A researcher, who was blinded to the study’s aims, measured the TCT for each suture performed by every participant. To ensure an objective evaluation of the time spent solely on suturing, TCT was defined as the duration from the moment the needle was grasped by the needle holder until the knot was cut with scissors. Additionally, 5 surgeons (7.2 [SD 1.7] years of surgical experience), who were also unaware of the study aims, evaluated suture performance based on anonymized photographs of performed sutures. All of the captured photographs of participants’ performed sutures were presented in a random order to evaluator surgeons using a custom visualization tool. They rated the performance of each suture type separately on a scale of 0 to 100, considering factors such as the overall effectiveness of the suture, bite (length of the stitch across the wound), pitch (interval between stitches), and cosmetic appearance [35].

### Study Task

**Figure 1.**
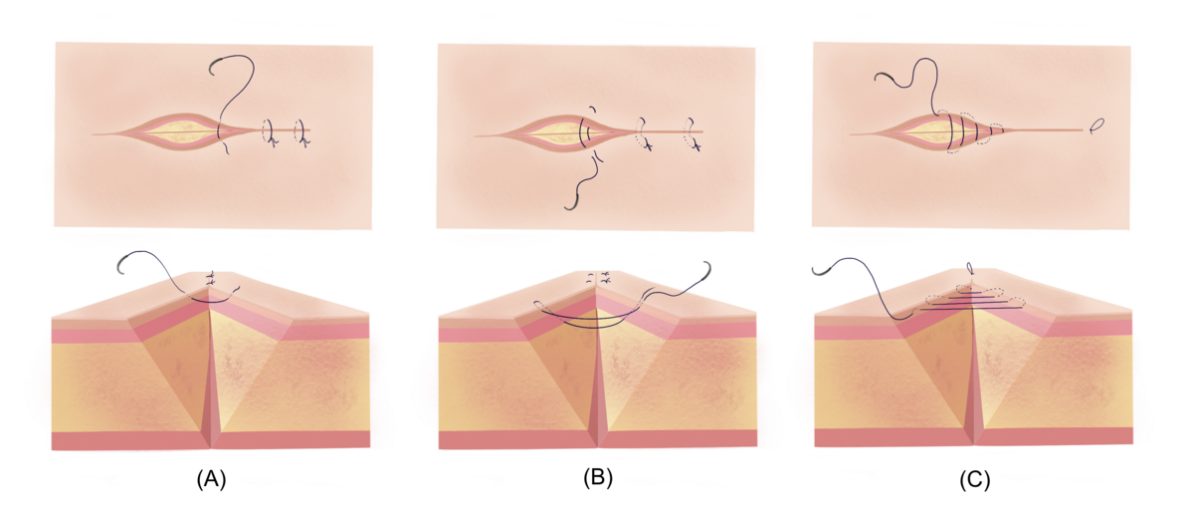
The illustration of 3 suture types included in the study task: (A) simple interrupted suture, (B) vertical mattress suture, and (C) continuous subcuticular suture.

To assess the feasibility of using AVP while performing precision-dependent medical applications and compare it with MHL2 and baseline (no HMD), we designed a controlled user study task that incorporates performing different suture techniques. Since the main goal of this study was to evaluate the usability of the device and its underlying technology without any bias from specific applications, no digital information was displayed in the HMDs used in this study (AVP and MHL2). The participants were instructed to perform the suturing task either using one of the HMDs or without any HMD in the baseline condition. This approach is particularly important for delicate tasks requiring fine motor control and precision in real-world applications such as surgery, where the device itself may affect task performance regardless of the usability of the XR application used. By isolating the device from application-related factors, this ensured an unbiased assessment of its usability, preventing findings from being influenced by app design or content. The study included 3 types of sutures, each requiring progressively more complex techniques, ranging from basic to advanced. The simple interrupted suture (SIS, Figure 1A) was selected as the simplest task, while the vertical mattress suture (VMS, Figure 1B) and continuous subcuticular suture (CSS, Figure 1C) were chosen for their complexity. These techniques rely on correct depth perception, as they involve inserting a suturing needle into a specific layer of the skin [35], making them ideal for evaluating users’ ability to perceive depth in a simulated environment.

**Figure 2.**
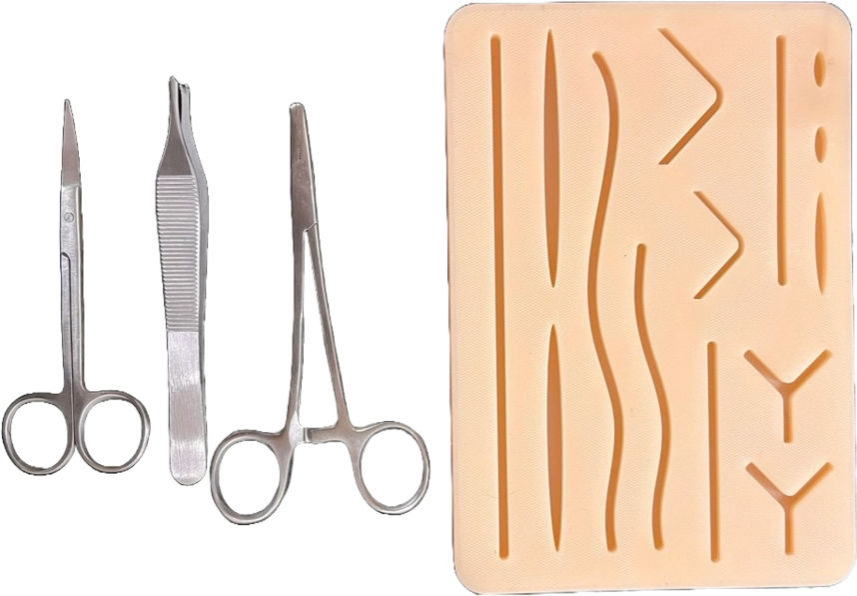
The suture training kit used during study tasks containing a silicone suture pad and instruments.

A suture training kit [36] was used as the base for performing the sutures. All participants were asked to complete 3 SIS, 3 VMS, and close a 5 cm long wound on the suturing kit using a CSS for each intervention. All sutures were performed using 3‐0 polypropylene [37] and the same clinical surgical instruments, including needle holder, tweezers, and scissors (Figure 2). To minimize potential bias in TCTs due to the length of the suture material, each suture type was performed with a new suture material.

### Participant Recruitment

Recruitment was conducted through word-of-mouth and advertisements via mail. The experiment took place at Klinikum Saarbrücken, Germany. Participation was entirely voluntary, and no compensation was provided.

The inclusion criteria for participants required that they be health care professionals with prior experience in performing wound suturing on patients. Additionally, the study limited participation to individuals without vision disorders or those with minor refractive errors, who could complete the study tasks without eyeglasses. To minimize bias, participants with minor refractive errors who typically used eyeglasses were instructed to perform all tasks without glasses across all interventions.

### Statistical Analysis

Sample size calculation was performed using the power analysis tool G*Power [38]. Since there is no previous work comparing participants’ performance using the AVP against MHL2 and baseline, we hypothesized a large between-group effect size (Cohen *f*) of 0.40 based on personal experience. This assumption was used to calculate the required sample size. With a power of 1–*β*=.95 and *α*=.05, the required sample size was calculated to be 18 participants. To account for 10% potential dropouts, we included a total of 20 participants in the study.

Statistical analysis was conducted using the R project for statistical computing [39]. Continuous data were expressed as means (SD), while categorical data were reported as frequencies and proportions. The effects of the interventions (baseline, MHL2, and AVP) were analyzed within-subject using repeated-measures ANOVA. When the assumption of sphericity was violated, the Greenhouse-Geisser (*ε*<.75) correction was applied. For the repeated measures ANOVA, we reported the F-statistic, degrees of freedom, *P* value, and generalized eta squared ($\eta_{G}^{2}$) as a measure of effect size. For post hoc pairwise comparisons, we conducted paired-samples *t* tests with Bonferroni correction for multiple within-group comparisons and reported the adjusted *P* values along with Cohen *d* to indicate effect size. A 2-sided *P* value of <.05 was considered statistically significant for all analyses. Furthermore, the inter-rater agreement of the performance scores by 5 evaluator surgeons was confirmed using the $r_{wG(J)}$ agreement index [40].

### Qualitative Analysis

All interviews conducted in this project were transcribed verbatim. We adopted a pragmatic approach to qualitative analysis, as recommended by Blandford et al [41]. Initially, 2 researchers independently analyzed the same 25% of the data. Based on iterative discussions, a preliminary coding framework was developed. The remaining 75% of the interview data were then evenly distributed between the 2 researchers for coding using the established coding framework. To further ensure the consistency between final codings in case of new code emergence or coding disagreements, in a final discussion, the coding framework was further refined, leading to the development of the main themes.

### Ethical Considerations

Before conducting the study, ethics approval was obtained from the institutional ethical review board of the German Research Center for Artificial Intelligence (DFKI, IRB approval number: VST – 48/25). All participants received comprehensive information about the objectives and data handling involved in this study. Data collection only proceeded after obtaining their voluntary informed consent. All participants were assured that their contributions would remain anonymous and were offered the opportunity to withdraw from the study at any stage prior to publication. All data were stored securely at the DFKI local server. Each participant signed a written consent form. No financial compensation was offered or provided.

## Results

### Study Population and Demographics

In total, 20 health care professionals participated in this study. The demographic characteristics of the participants are detailed in Table 1.

**Table 1.** Demographics of study participants.

Characteristics	Value
Gender, n (%)	
Man	14 (70)
Woman	6 (30)
Age (years), mean (SD)	33.65 (7.60)
Occupation, n (%)	
Surgeon	15 (75)
Physician assistant	1 (5)
Medical intern	4 (20)
Participants with minor refractive errors, n (%)	6 (30)
Clinical experience (years), mean (SD)	7.8 (6.45)
Prior use of OST^a^ HMDs^b^ (1‐5 Likert scale), mean (SD)	1.8 (0.93)
Prior use of VST^c^ HMDs (1‐5 Likert scale), mean (SD)	1.35 (0.63)
Affinity for technology interaction (1‐6 Likert scale), mean (SD)	3.83 (0.79)

a OST: optical see-through.

b HMD: head-mounted display.

c VST: video-see-through.

### User Performance

#### Suturing Performance

**Figure 3.**
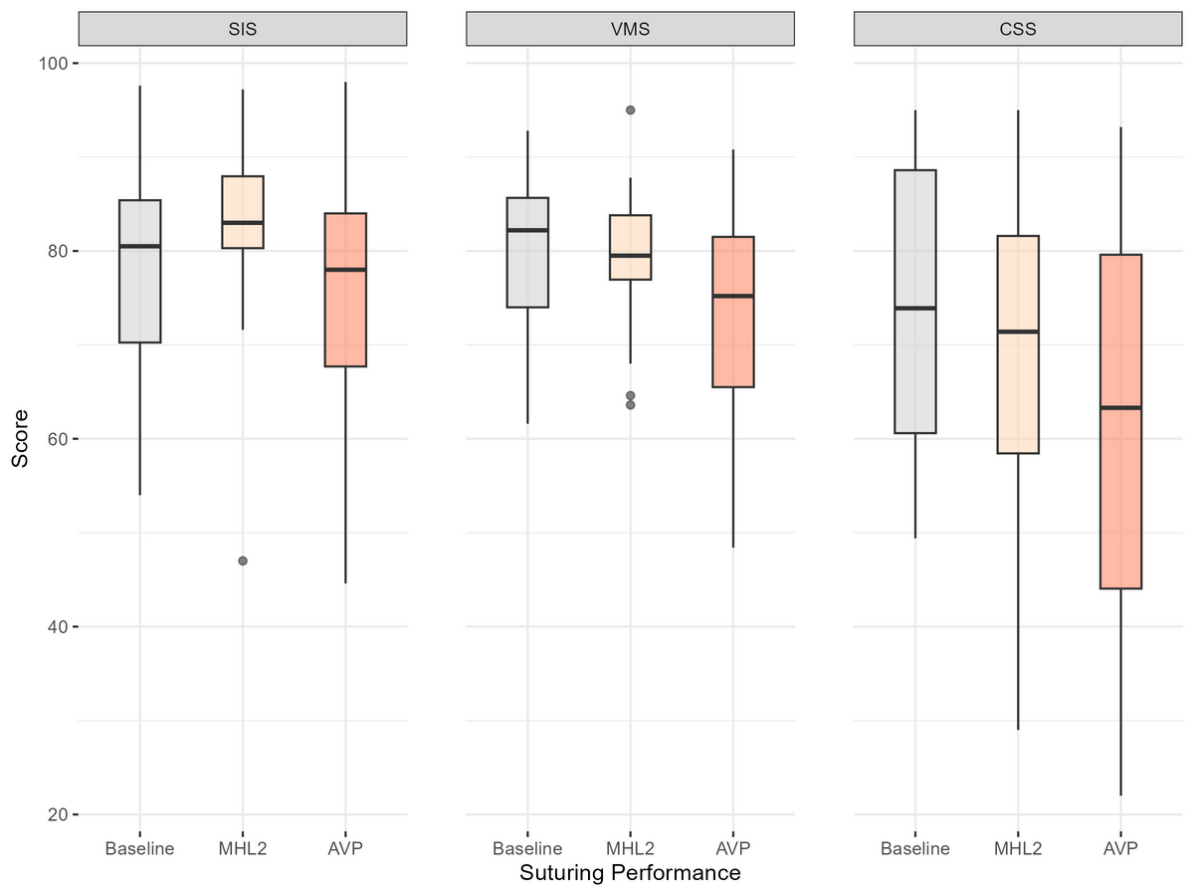
The participants’ suturing performance rated by surgeons across different suture types. AVP: Apple Vision Pro; CSS: continuous subcuticular suture; MHL: Microsoft HoloLens; SIS: simple interrupted suture; VMS: vertical mattress suture.

The 5 surgeons’ evaluation scores of participants’ suture performances showed high agreement, with an $r_{wG(J)}$ value greater than 0.99 across the suture types. The analysis on participants’ average suture performance revealed no significant differences across interventions (baseline=73.8 [SD 13.5], AVP=74.5 [SD 10.0], MHL2=75.3 [SD 9.7], *F*_2, 38_=0.28, *P*=.76, $\eta_{G}^{2}$=0.003). Despite the worsened performance with AVP for all suture types, no significant differences were observed between interventions for any suture type (Figure 3).

#### Task Completion Time

The analysis revealed a significant difference in the TCT required to complete all tasks across the interventions (baseline=472.0 [SD 143.0] s, AVP=570.0 [SD 192.0] s, MHL2=456.0 [SD 120.0] s; *F*_2, 38_=17.6, *P*<.001, $\eta_{G}^{2}$=0.101). Pairwise test results showed participants took significantly longer to complete the entire task using AVP to MHL2 (*P*<.001, Cohen *d*=1.02, large effect) and baseline (*P*<.001, Cohen *d*=1.03, large effect). The comparison between baseline and MHL2 showed a small effect size (*P*=.30, Cohen *d*=0.2, small effect), suggesting minimal difference.

**Figure 4.**
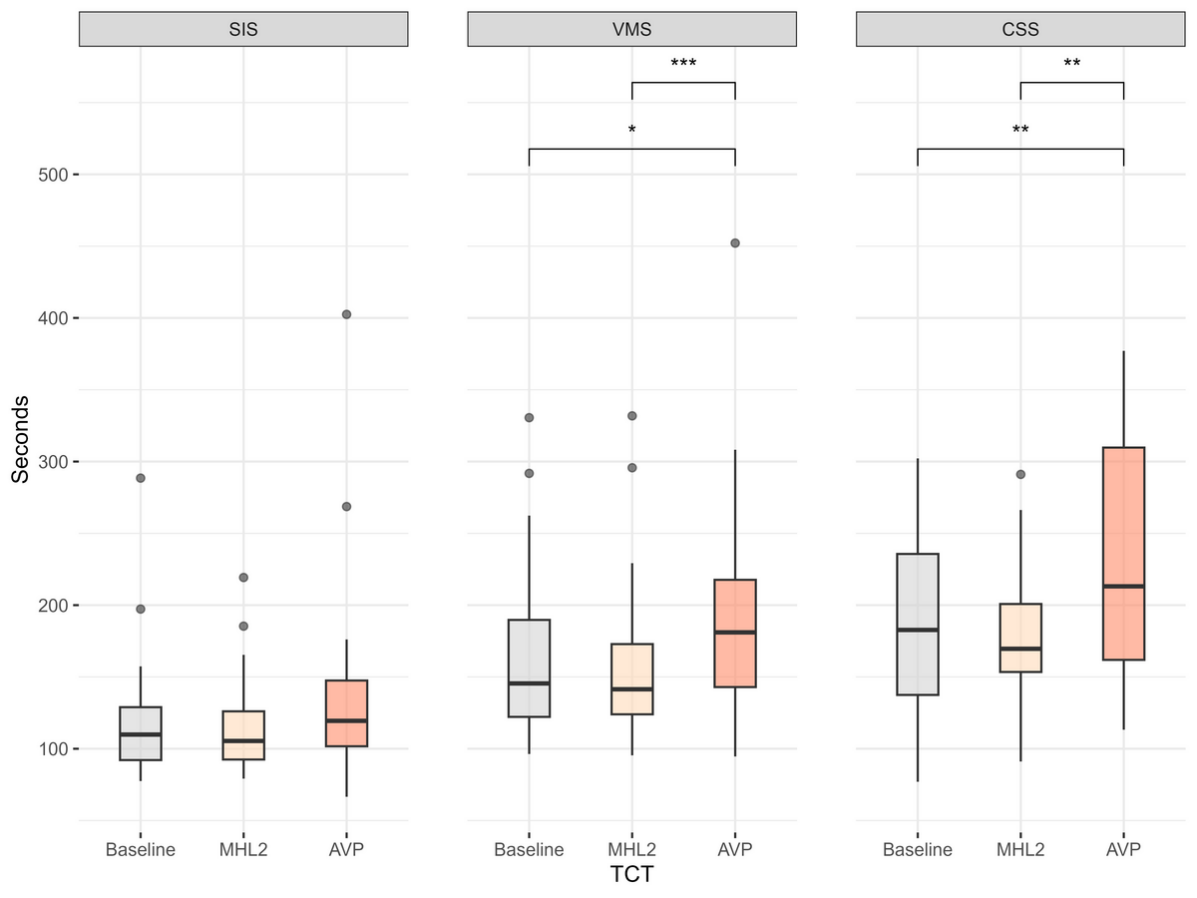
Participants’ TCTs across different suture types. Statistically significant differences are denoted as follows: *P<.05, **P<.01, and ***P<.001. AVP: Apple Vision Pro; CSS: continuous subcuticular suture; MHL: Microsoft HoloLens; SIS: simple interrupted suture; TCT: task completion time; VMS: vertical mattress suture.

Analysis performed on TCT for each individual suture type (Figure 4) revealed that there were no significant differences in the time required to perform SIS across the interventions (baseline=122.2 [SD 48.9] s, AVP=139.8 [SD 76] s, MHL2=117.5 [SD 37.5] s; *F*_1.4, 27.0_=3.67, *P*=.05, $\eta_{G}^{2}$=0.03). However, a significant difference was observed for more complex tasks, VMS (baseline=166.8 [SD 64.2] s, AVP =195.9 [SD 79.6] s, MHL2=161.2 [SD 63.4] s; *F*_1.5, 28.8_=9.66, *P*=.001, $\eta_{G}^{2}$=0.048) and CSS (baseline=182.9 [SD 59.1] s, AVP =234.7 [SD 83.6] s, MHL2=177.0 [SD 52.8] s; *F*_1.4, 27.3_=11.7, *P*<.001, $\eta_{G}^{2}$=0.138).

Pairwise test results showed participants required significantly more time to complete VMS using AVP compared with MHL2 (*P*<.001, Cohen *d*=1.0, large effect) and baseline (*P*=.04, Cohen *d*=0.6, moderate effect). The comparison between baseline and MHL2 showed no significant difference (*P*=.99, Cohen *d*=0.2, negligible effect).

Similarly, for CSS, a significantly longer time was needed when using AVP compared with MHL2 (*P*=.007, Cohen *d*=0.8, moderate effect) and baseline (*P*=.002, Cohen *d*=0.9, large effect). No significant difference was observed between baseline and MHL2 (*P*=.99, Cohen *d*=0.1, negligible effect).

### User Experience

#### Cognitive Workload

The analysis revealed a significant difference in the total raw NASA-TLX score across the interventions (baseline=19.1 [SD 15.1], AVP=43.9 [SD 15.9], MHL2=21.5 [SD 13.8]; *F*_1.2, 21.9_=28.6 *P*<.001, $\eta_{G}^{2}$=0.37). The pairwise test results showed the total raw NASA-TLX score among participants was significantly higher for AVP compared with MHL2 (*P*<.001, Cohen *d*=1.2, large effect) and baseline (*P*<.001, Cohen *d*=1.3, large effect). No significant difference was observed between baseline and MHL2 (*P*=.30, Cohen *d*=−0.401, negligible effect).

**Figure 5.**
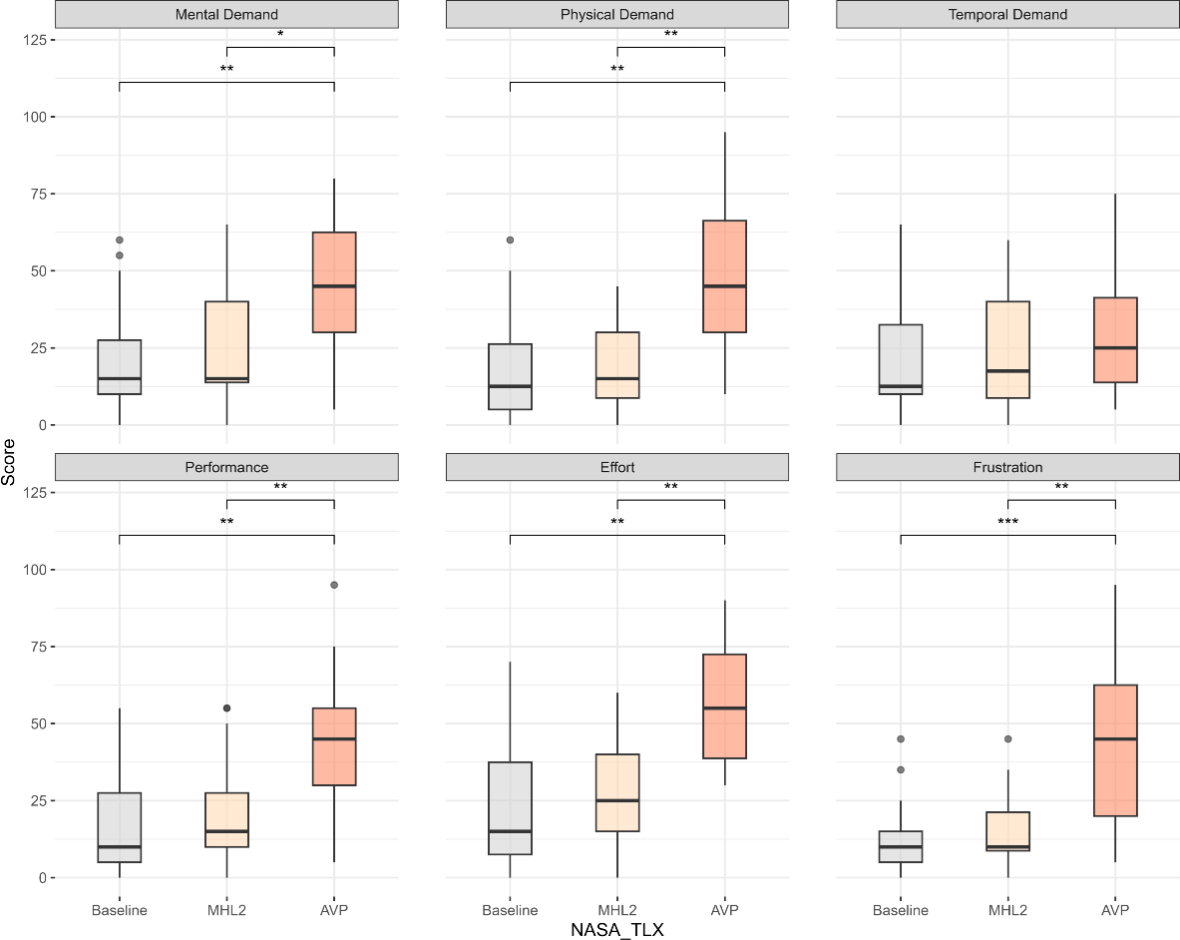
NASA-TLX results for each factor (mental demand, physical demand, temporal demand, performance, effort, and frustration) across the 3 interventions. Statistically significant differences are denoted as follows: *P<.01, **P<.001, and ***P<.0001. AVP: Apple Vision Pro; MHL: Microsoft HoloLens; NASA-TLX: NASA Task Load Index.

Analysis on each scale of NASA-TLX showed significant differences on mental demand (baseline=20.2 [SD 18.0], AVP=46.0 [SD 22.9], MHL2=23.75 [SD 18.6]; *F*_1.2, 22.8_=21.1, *P*<.001, $\eta_{G}^{2}=0.256$), physical demand (baseline=17.2 [SD 17.3], AVP =46.0 [SD 25.0], MHL2=19.2 [SD 14.3]; *F*_1.1, 21.3_=22.4, *P*<.001, $\eta_{G}^{2}$=0.324), performance (baseline =18.2 [SD 16.5], AVP =45.5 [SD 21.2], MHL2=20.7 [SD 16.6]; *F*_1.2, 22_=3.6, *P*<.001, $\eta_{G}^{2}=0.3$), effort (baseline=24.5 [SD 23.4], AVP =56.0 [SD 20.0], MHL2=28.0 [SD 18.0]; *F*_1.3, 24.5_=23.4, *P*<.001, $\eta_{G}^{2}$ =0.3), and frustration (baseline=12.25 [SD 11.7], AVP=43.5 [SD 23.4], MHL2=15 [SD 12.1]; *F*_1.1, 21_=26.1, *P*<.001, $\eta_{G}^{2}$=0.4) factors. Pairwise analysis showed significantly higher scores for AVP compared with MHL2 for mental demand (*P*<.01, Cohen *d*=1.0, large effect), physical demand (*P*<.001, Cohen *d*=1.2, large effect), performance (*P*<.001, Cohen *d*=1.0, large effect), effort (*P*<.001, Cohen *d*=1.1, large effect), and frustration (*P*<.001, Cohen *d*=1.1, large effect). Similarly, significant differences were observed for AVP compared with baseline on mental demand (*P*<.001, Cohen *d*=1.1, large effect), physical demand (*P*<.001, Cohen *d*=1.0, large effect), performance (*P*<.001, Cohen *d*=1.1, large effect), effort (*P*<.001, Cohen *d*=1.1, large effect), and frustration (*P*<.001, Cohen *d*=1.2, large effect). No significant difference was observed between MHL2 and baseline on any of the NASA-TLX factors (Figure 5).

#### Presence

The analysis of PQ demonstrated a significantly higher presence score (AVP=93.7 [SD 12.7], MHL2=115.0 [SD 11.4]; *F*_1, 19_=27.9, *P*<.001, $\eta_{G}^{2}$=0.4) for MHL2 compared with AVP. This significant difference was observed in all factors of presence including the realism (AVP=34.2 [SD 4.9], MHL2=41.0 [SD 5.3]; *F*_1, 19_=17.2, *P*<.001, $\eta_{G}^{2}$=0.3), possibility to act (AVP=20.6 [SD 3.5], MHL2=25.5 [SD 2.8]; *F*_1, 19_=19.8, *P*<.001, $\eta_{G}^{2}$=0.4), quality of interface (AVP=14.8 [SD 3.5], MHL2=18 [SD 3.0]; *F*_1, 19_=19.7, *P*<.001, $\eta_{G}^{2}$=0.2), possibility to examine (AVP=13.9 [SD 3.6], MHL2=18.3 [SD 2.3]; *F*_1, 19_=20.1, *P*<.001, $\eta_{G}^{2}$=0.4), and self-evaluation of performance (AVP=10.2 [SD 2.8], MHL2=12.2 [SD 2.0]; *F*_1, 19_=5.5, *P*=.03, $\eta_{G}^{2}$=0.1).

#### VR Sickness

The overall VRSQ score (AVP=66.9 [SD 19.8], MHL2=41.1 [SD 9.32]; *F*_1, 19_=46.7, *P*<.001, $\eta_{G}^{2}$=0.4) was also significantly higher for AVP compared with MHL2. The participants rated significantly higher scores on both oculomotor (AVP=75.8 [SD 22.9], MHL2=42.9 [SD 12.8]; *F*_1, 19_=69.8 *P*<.001, $\eta_{G}^{2}$=0.4) and disorientation (AVP=58 [SD 19.0], MHL2=39.3 [SD 9.15]; *F*_1, 19_=18.9, *P*<.001, $\eta_{G}^{2}$0.3) factors for AVP compared with MHL2.

#### System Usability

System usability score for MHL2 was significantly higher compared with AVP (AVP=50.3 [SD 14.4], MHL2=72.7 [SD 8.54]; *F*_1, 19_=34.4, *P*<.001, $\eta_{G}^{2}$=0.5).

#### Interviews

##### Overview

After thematic analysis of the interviews, we developed 5 main themes: comfort and physical strain, visual challenges and depth perception, self-evaluation of performance, user confidence and preference, and application domain.

##### Comfort and Physical Strain

Thematic analysis of observations made during the study revealed several comfort-related issues associated with AVP. Participants reported discomfort due to the unbalanced weight distribution, with the majority of the weight concentrated on the nasal and maxillary area, leading to strain in the back neck muscles and headaches. Two participants commented on this with the following statements:

*When you look straight it is more convenient but when you bend your head to look at the stitch pad which I think would be the normal case when you operate around the table, then it is too uncomfortable, because the whole weight is in front and there is a constant contraction on your neck.*

[P9, surgeon]

*I usually get VR sickness whenever I use VR headsets. I tested AVP before and previously didn’t have any issues when watching videos and so on, but here I got a very bad headache because I think I tried too much to focus and finish my task.*

[P1, surgeon]

Additionally, they described a sensation of instability when focusing on a task for an extended period, expressing that it felt as though their head might fall forward.

*It [AVP] is very heavy and after a while you feel like your head would fall down if you don’t consistently fight it. And I can assure you it was a relief to take it out.*

[P13, surgeon]

In contrast, no incidents of discomfort were observed with MHL2 for the period the participants used MHL2 for the experiment. One of the participants also indicated that the design of MHL2 is more comfortable and suited for use in the operating room compared with AVP.

*We didn’t use it here, but I guess with HoloLens you have also this option where you could push the visor up or down based on what you want to see but with AVP you don’t even have that option. You just have to take it off completely. And it is just not practical to use it during operation if you have to take it off every time.*

[P1, surgeon]

##### Visual Challenges and Depth Perception

Participants also experienced visual challenges with AVP, including blurred vision and difficulties with depth perception. The struggle with depth perception further impacted their ability to accurately judge distances, which is critical for suturing.

*My vision felt a bit blurry; I could not see the details I had difficulties to see the needle and also to do the knots.*

[P12, surgeon]

*For the first two sutures AVP was also ok but for the last suture type you have to really see where you put your needle in and that was simply impossible to make sure you are in the correct layer.*

[P11, physician assistant]

*I don’t think that my hand and eye coordination was disturbed, but it was very difficult to estimate the depth, there was like less contrast compared to reality.*

[P4, surgeon]

Participants also reported that the sharpness of the view varied depending on the distance of the objects from them. Some noted that maintaining a greater distance provided a sharper view; however, this was not ideal for delicate tasks, as they naturally tended to lean in for a closer observation and better precision.

*I could see things clearly farther than one meter to me. I could even read small letters, but when I looked at the stitching pad which was closer to me then it became blurry. And got even blurrier when I was leaning closer to it to do the stitches. Which usually you should see better when you get closer, but it just made it worse.*

[P13, surgeon]

##### Self-Evaluation of Performance

Some participants believed that AVP negatively impacted their performance compared with MHL2 and baseline.

*I used AVP before, but I only used it to watch videos, and initially I thought AVP would be better compared to HoloLens, but it was a complete catastrophe. I almost saw nothing. Yes, I did sutures from experience, but it was a complete guess work especially for the last suture type.*

[P1, surgeon]

*Naturally you realize a difference between no glass and having glass for both devices. But the difference was simply too much for AVP that I think it really impacted my performance.*

[P11, physician assistant]

*The HoloLens was not a big influence in my performance compared to performing without one it felt like having a light shaded sunglass on. If you look through the screen the vision is a bit darker but doesn’t make your performance worse. AVP is very immersive but for precise work like stitches it’s not fast enough and the resolution is not optimal.*

[P4, surgeon]

##### User Confidence and Preference

Participants expressed a preference for OST over VST. They reported that VST created a sense of disconnection from the real world, which was also evident in the lower presence score compared with OST. They reported that in real-life scenarios involving patients, it would impact their confidence. In contrast, OST allowed them to maintain situational awareness and benefit from a wider peripheral vision, enhancing their overall experience and performance. Participants reflected on this, saying:

*With HoloLens I felt more secure, because I think my peripheral vision was not affected that much but in AVP even though you still see but you have more restricted peripherals.*

[P2, surgeon]

*I think optimally the best is no glass but if I should choose, I think when you talk to a patient or your colleagues around the surgery table it feels just more natural to have eye contact even if it is through a glass like this [showing MHL2]. It is better than having a big headset on your face where no one can see your eyes in there. It is just more assuring with a see-through glass than a completely closed one [AVP].*

[P18, surgeon]

##### Application Domain

The participants regarded MHL2 as a usable device for various medical applications, including applications for intraoperative use. However, they believed that AVP would be more suitable for domains such as training or surgical planning, where the device would not be used during actual patient operations.

*I think both devices could be used for medical domain, but I won’t feel comfortable operating with the first one [AVP]. I think it is risky if you operate on veins or arteries. I don’t want to take any risk when operating on a patient.*

[P2, surgeon]

*I see HoloLens as a usable device during operation, it won’t stress you, but AVP would be a better fit for training or teaching or perhaps surgery planning.*

[P8, surgeon]

A participant also suggested that AVP could be used for surgical applications, such as laparoscopic surgery, where the surgical field is already viewed through high-resolution video.

*I think AVP would be useful for laparoscopic surgery where your view to the operation scene is already through a video and with AVP you can have this high-quality video stream.*

[P1, surgeon]

## Discussion

### Overview

With advancements in computational power, camera technology, and display systems, a noticeable trend is emerging in commercially produced HMDs. Manufacturers are increasingly shifting from dedicated AR and VR HMDs toward XR HMDs capable of supporting both functionalities. This transition is also evident in the evolution of recent HMDs developed by well-known brands, such as Apple [3] and Meta [42], which use VST displays, in contrast to earlier designs like Microsoft’s HoloLens [13], which relied on OST technology. Although this transition is expected to bring advantages beyond simply combining AR and VR, such as a wider field of view, higher camera quality, brightness control, and ultimately more precise spatial registration of digital objects, its feasibility in domains requiring high precision, such as intraoperative use, remains untested. Despite the foreseen potential benefits that recent XR glasses could bring to the medical domain [4,5,12], their feasibility in high-precision medical applications remained an open question. While most related studies focus on evaluating specific immersive applications within this domain [7,28,43], the choice of used devices is often driven by market trends rather than a critical assessment of their suitability for the intended use. Although some studies have compared the technical capabilities of different HMDs, including VST and OST design [44-46], they often overlook user experience and performance outcomes.

The findings of this study underscore the importance of device selection, particularly in time-sensitive and precision-dependent medical contexts. As progressively more immersive HMDs are being produced, our results demonstrate that the appropriateness of the chosen device itself plays a pivotal role in the user’s real-world performance—even before any application is introduced. Neglecting to assess the suitability of the device as an initial step may contribute to the negative user experience and delayed integration of immersive technologies in clinical settings, as the hardware itself may be ill-suited for the domain—even when the application might offer substantial potential benefits.

### Principal Results

In this study, we evaluated the feasibility of AVP (the most recent and promising XR HMD for the medical domain [10,12]) with MHL2, the commonly used MR HMD in the medical domain for precision tasks [14-22,24,25,28]. Twenty health care professionals participated in the study, performing suture tasks under 3 conditions: AVP, MHL2, and a baseline condition without an HMD. We evaluated user performance and experience across these conditions. Post hoc analysis of the primary outcome measures revealed a large observed effect size between AVP, MHL2, and the baseline, suggesting that the sample size (n=20) was adequate and confirming the validity of our initial power analysis. Our findings highlight the distinct strengths of each HMD, suggesting their suitability for different medical use cases.

A key observation from our study was the significant increase in cognitive load when using AVP compared with MHL2 and the baseline condition. This higher cognitive burden was accompanied by increased TCTs, indicating that AVP demands a greater cognitive workload from users. Interestingly, despite these challenges, objective performance evaluations did not show significant differences across the interventions. However, participants’ self-reported performance, as captured in the PQ, indicated a significant decline compared with MHL2. This divergence between expert-assessed performance scores and self-evaluations may reflect a reduction in participants’ confidence in their task performance, a sentiment that was also echoed in the interview responses.

These findings suggest that AVP remains a feasible option for non-time-sensitive medical domains where cognitive workload and TCT are not critical factors, such as a surgical planning tool demonstrated by Olexa et al [7], where there is no trade-off between the benefits of the used application and the need for real-world precision and speed.

Moreover, AVP potentially stands as a feasible device for medical education and training, incorporating virtual reality simulations where interaction with digital elements is prioritized over real-world precision. However, the usability of AVP while training precision tasks in the real world might be limited. A participant’s sentiment on performing sutures from experience instead of relying on their visual perceptions indicates that training real-world precision tasks with AVP could potentially cause negative impacts on the learning process. A device that inadvertently increases cognitive demand or induces disorientation, as observed with AVP (Figure 5), might lead to suboptimal skill acquisition, potentially compromising the training outcomes. In educational contexts, the choice of XR technology can significantly influence learning behavior. Although positive outcomes have been demonstrated in nonmedical areas such as design education [47] over nonimmersive devices, the choice of immersive devices should still be carefully considered in educational settings, particularly those that emphasize psychomotor skills. An educational XR system that imposes excessive cognitive workload or fails to foster a strong sense of presence may lead to the development of maladaptive motor patterns or “incorrect” muscle memory, ultimately impacting long-term real-world performance.

Furthermore, AVP could serve as a practical solution for applications where direct real-world perception is either not required or is transmitted to the device instead of being captured by it, such as in telemedicine. Similarly, in fields where user presence is essential but critical information is traditionally delivered through digital interfaces, like laparoscopic surgery, where surgeons view the operative field on a 2D monitor, the potential of AVP could be further explored and leveraged. However, for use cases that involve fine motor tasks, such as those suggested by previous works including medical training scenarios [10,48] or in more critical applications such as intraoperative support tools [48], the increased workload and disorientation associated with AVP might hinder user real-world performance. Conversely, MHL2 demonstrated its suitability for applications requiring high precision and time efficiency. Its OST design allowed for a lower cognitive load and better user experience during the suturing task, with no significant differences when compared with the baseline. This underscores its potential for intraoperative use cases, such as surgical navigation and other real-time assistance tools, where maintaining a strong connection to the real world is critical.

Unlike the findings of the study by Olexa et al [7], where participants reported minimal eye strain or fatigue, our questionnaire responses on VR sickness indicated heightened oculomotor strain and disorientation with AVP. This discrepancy could be attributed to differences in user attention directed toward digital versus real-world objects. In the study by Olexa et al [7], users primarily focused on digital objects, whereas in our study, the main focus was on real-world perception. This phenomenon was also corroborated by one of our participants, who had prior experience using AVP. In our study, participants frequently reported that AVP caused physical discomfort—including eye strain, headaches, and neck fatigue—as well as visual challenges such as blurriness, difficulty focusing, and disorientation during head movement. These sensory and ergonomic limitations often led to reduced task confidence and greater reliance on prior experience or instinct rather than real-time visual feedback. In contrast, MHL2 was consistently described as lightweight, comfortable, and minimally intrusive, with clearer visual output and fewer disruptions to the user’s natural workflow. Such qualitative insights underscore the critical role of comfort and visual clarity in sustaining task engagement and motor coordination over time. These human-centered considerations are especially relevant in domains where extended use or precision is essential. AVP, in its current form with display quality and ergonomic constraints, may be more suitable for fully immersive VR applications rather than AR/MR-integrated medical use, particularly for shorter durations. In contrast, MHL2 demonstrated lower VR sickness scores, attributed to its OST design, along with a more balanced weight distribution and higher presence scores. These features position MHL2 as better optimized for applications requiring extended use periods and seamless real-world connectivity.

The findings of this study highlight several factors that could inform the future design of XR and MR devices for the medical domain. While generalizability remains a goal for widespread adoption across diverse medical applications, custom designs may be more appropriate for time-critical use cases, such as surgical navigation systems. The optimal approach may vary depending on the method used to visualize the surgical scene, whether open surgery, laparoscopic, or robotic-assisted procedures. For open surgery, OST displays could provide distinct advantages by preserving a clear view of the real world and facilitating seamless communication with the surgical team. Conversely, in scenarios involving indirect surgical views, such as laparoscopic procedures, VST HMDs might offer greater benefits. Furthermore, the future integration of VST HMDs in precision-demanding applications requires high-quality camera feed from a real-world environment; challenges such as camera focus issues, which result in blurring of the real-world video stream, can significantly hinder the usability of these devices in tasks requiring precision. Finally, ensuring comfort during extended use is critical for intraoperative tools. Features such as balanced weight distribution, antimicrobial coatings, and easy-to-clean surfaces would further facilitate smoother integration into clinical workflows.

### Limitations

While this study provides valuable insights, it also has several limitations. First, we focused solely on evaluating the feasibility of AVP for medical precision tasks and compared the outcomes with MHL2 as a representative example of existing MR devices, given its extensive prior use in the medical domain. To further validate the generalizability of our findings regarding comparisons between various VST and OST displays, additional research involving other available HMDs is necessary. Second, our study included only participants with no or minor refractive errors who were able to complete the tasks without eyeglasses. To minimize bias, the 6 participants with minor refractive errors were asked to perform all tasks across all interventions without wearing eyeglasses. Although it is possible to wear eyeglasses with MHL2, this approach was not feasible for AVP due to its design. While there is an option to integrate correction lenses into AVP, customizing lenses for each participant was impractical and not feasible. Finally, while AVP is expected to enhance the display of digital objects, no digital elements were incorporated into the study tasks, as this was beyond the scope of our research. Our primary objective was to assess the safety and feasibility of AVP as a VST-HMD for performing medical precision tasks.

### Conclusions

In conclusion, while AVP shows promise for non-time-sensitive applications that do not have an emphasis on real-world perception, MHL2 remains the preferred choice for time-critical and precision-demanding tasks. Further research and device refinements will be necessary to fully integrate XR HMDs into diverse medical applications, ensuring both user comfort and operational efficiency.

## Abbreviations

AR: augmented reality
AVP: Apple Vision Pro
CSS: continuous subcuticular suture
HMD: head-mounted display
MHL: Microsoft HoloLens
MR: mixed reality
NASA-TLX: NASA Task Load Index
OST: optical see-through
PQ: presence questionnaire
SIS: simple interrupted suture
TCT: task completion time
VMS: vertical mattress suture
VR: virtual reality
VRSQ: virtual reality sickness questionnaire
VST: video-see-through
XR: extended reality
